# The Role of Hyaluronan and CD44 in the Pathogenesis of Lupus Nephritis

**DOI:** 10.1155/2012/207190

**Published:** 2012-08-01

**Authors:** Susan Yung, Tak Mao Chan

**Affiliations:** Department of Medicine, University of Hong Kong, Room 302 New Clinical Building, Queen Mary Hospital, Pokfulam, Hong Kong

## Abstract

Systemic lupus erythematosus (SLE) is a prototype autoimmune disease that affects multiorgan systems. Lupus nephritis is one of the most severe manifestations of SLE whereby immune-mediated inflammation can lead to permanent damage within the glomerular, tubulo-interstitial, and vascular compartments of the kidney, resulting in acute or chronic renal failure. The mechanisms that regulate host inflammatory responses and tissue injury are incompletely understood. Accumulating evidence suggests that hyaluronan and its interaction with its cell surface receptor CD44 plays an important role in mediating pathogenic mechanisms in SLE. This paper discusses the putative mechanisms through which hyaluronan and CD44 contribute to the pathogenesis of SLE, with particular emphasis on lupus nephritis.

## 1. Introduction

Systemic lupus erythematosus (SLE) is a severe autoimmune disease characterized by a breakdown of immune tolerance and production of autoantibodies. Although the etiology of SLE remains to be fully elucidated, accumulating evidence suggests that genetic, environmental, infectious, and hormonal factors may predispose individuals to the development of SLE [1–3]. This disease predominantly affects females of Afro-American, Hispanic, and Asian descent and can be mild or life threatening depending on the organs involved. 

Renal involvement occurs in up to 60% of SLE patients and is a strong predictor of morbidity and mortality [4]. Onset of lupus nephritis is initiated by the deposition of anti-double stranded (ds) DNA antibodies in the kidney parenchyma, which results in complement activation, infiltration of immune cells, and induction of inflammatory and fibrotic processes in the kidney. If these tissue-damaging processes are not sufficiently controlled, destruction of the normal kidney parenchyma and its replacement by fibrous tissue will ensue, which will lead to endstage renal failure [4]. The exact mechanisms through which anti-dsDNA antibodies are deposited in the kidney to mediate kidney injury remains to be fully defined but current knowledge suggests that they can bind directly to mesangial cells through annexin II or *α*-actinin [5–7] or indirectly to components of the glomerular basement membrane through nucleosomes [8, 9]. 

The extracellular matrix (ECM) was previously considered to function solely as a structural support that maintained the architecture of tissues and organs, but there is now compelling evidence to show that ECM components also play critical roles during inflammatory processes. Their accumulation and subsequent degradation is a cardinal feature of autoimmune diseases. Hyaluronan (HA) is a major component of the ECM that can directly regulate inflammatory processes through its interaction with CD44, its cell surface receptor [10, 11]. Depending on its molecular weight HA may possess either anti-inflammatory or pro-inflammatory properties. We have demonstrated that serum HA levels in patients with lupus nephritis correlate with disease activity, and that intrarenal HA expression is also increased in lupus nephritis, induced in part by anti-dsDNA antibodies [12]. This paper will discuss the putative roles of HA and CD44 in SLE, with particular emphasis on their roles in mediating inflammatory processes during lupus nephritis.

## 2. Hyaluronan and CD44

### 2.1. Synthesis of HA

HA is a nonsulfated, negatively charged glycosaminoglycan that is composed of repeating disaccharide units of D-glucuronic acid and N-acetyl-D-glucosamine [13]. Unlike other glycosaminoglycans, HA is not attached to a protein core and is synthesized on the inner surface of the plasma membrane [13]. HA is synthesized by HA synthases (HAS) and currently three mammalian HAS have been identified, namely, HAS I, HAS II, and HAS III, which utilize UDP-*α*-N-acetyl-D-glucosamine and UDP-*α*-glucuronate as substrates for the synthesis of HA [14]. Under physiologic conditions, HA is synthesized as a macromolecule with a MW of 10^5^–10^7^ Da depending on the tissue type [15]. Following its synthesis, HA is directed to the cell surface where it interacts with CD44, or is assembled into pericellular or extracellular matrices [16]. Studies have demonstrated that all three HAS isozymes can contribute to the synthesis of high MW (HMW) HA, but HAS I and HAS III may also produce low MW (LMW) HA depending on the condition of the microenvironment [14].

### 2.2. Functions of HA under Physiological and Pathological Conditions

Despite its simple chemical structure, HA remains one of the most complex and multifaceted components of the ECM that contributes to diverse biological functions such as the structural stability of basement membranes, maintenance of water balance, plasma protein distribution, sequestration of free radicals, and regulation of cell proliferation, migration, and phenotype [17]. Native HA possesses anti-inflammatory, anti-angiogenic, and immunosuppressive properties [18]. They also provide a protective glycocalyx around endothelial, epithelial, and mesothelial cells that protect these cells from injury, apoptosis, and leukocyte adhesion [19–21]. HA undergoes constant turnover during the daily maintenance of basement membranes. It is degraded into small, nonbiologically active fragments which is rapidly removed through the liver. 

The turnover and remodeling of the ECM is a dynamic process that occurs during normal development and tissue repair, and replenishment of ECM components is critical in order to preserve the structural and functional integrity of tissues. These processes become aberrant in pathological conditions associated with chronic inflammation where accumulation of ECM constituents is often observed, which perturbs tissue structure resulting in organ dysfunction. HA accumulates at sites of injury during chronic renal inflammation, where they form long cable-like structures that act as an adhesive matrix for the binding of leukocytes and macrophages. Mesangial cells and proximal tubular epithelial cells have been shown to contribute to the synthesis of these cable-like structures [22, 23]. In line with its anti-inflammatory properties, it has been suggested that binding of leukocytes to HA cables prevents them from interacting with adhesion molecules, thus limiting inflammatory processes in the glomerulus and tubulo-interstitium. Furthermore, it is also conceivable that the HA cable may serve as a temporary scaffold that prevents the loss of ECM components during extreme tissue remodeling [24]. Macrophages have been shown to regulate the clearance of the provisional HA matrix, and this process is essential before a permanent matrix can be synthesized. 

Unlike other glycosaminoglycans where modifications in their sulfation pattern, deacetylation and epimerization define their biological roles, the functional role in HA is dictated by its molecular weight and its interaction with its binding proteins, the latter termed the hyaladherins. HA undergoes depolymerization either through oxidative stress or enzymatic cleavage by various hyaluronidases during tissue injury and inflammatory processes [10, 11, 25]. LMW HA have biological properties that are distinct from their parent molecule and have been shown to promote inflammatory and angiogenic processes through increased cell proliferation, activation of signaling transduction pathways and induction of chemokine and cytokine secretion in macrophages, dendritic cells, mesothelial cells, mesangial cells, epithelial cells, and chondrocytes [10, 26–32]. The clearance of HA fragments is therefore imperative for the resolution of tissue injury. The removal of LMW HA from sites of injury is dependent on their interaction with CD44 since targeted deletion of CD44 in mice with bleomycin-induced lung injury resulted in the accumulation of HA fragments, unremitting inflammation, and perpetual tissue damage, a finding that was not observed in wild-type mice [33]. The distinct biological roles of HMW and LMW HA thus far identified are summarized (Table 1). An in-depth review of the interaction of HA with hyaladherins and mechanisms of degradation is outside the scope of this paper [10, 11, 13, 16].

### 2.3. CD44

**Figure 1:**
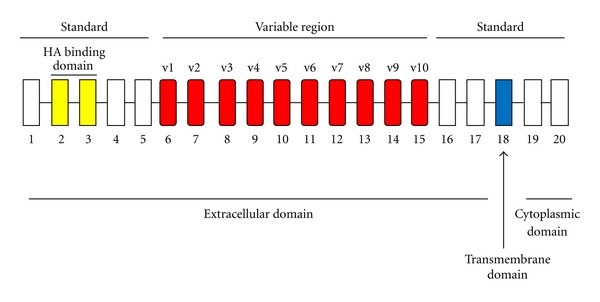
Genomic structure of CD44. The gene encoding for human CD44 consists of 20 exons. The standard form of CD44 contains exons 1–5, 16–18, and 20. Variants forms of CD44 comprise the standard form of CD44 and the insertion of various combinations of variant exons (v1–v10). Exon 19 is normally absent in most CD44 transcripts and its inclusion results in a shorter variant form of CD44.

CD44 is a transmembrane glycoprotein with a wide tissue distribution and is found on leukocytes, and epithelial, endothelial, and smooth muscle-like cells. The human CD44 gene is located on the short arm of chromosome 11 and consists of 20 exons of which 10 are variant exons (v1–v10) that can undergo alternative splicing to generate multiple CD44 isoforms [34]. The genomic structure of CD44 is shown in Figure 1. Post-translational modifications of the CD44 molecule such as N- and O-glycosylation, and the attachment of heparan sulfate and/or chondroitin sulfate glycosaminoglycan chains may further increase the number of CD44 isoforms. Such post-translational modifications are tissue specific and bestow upon the CD44 molecule an ability to sequester growth factors and cytokines, thereby allowing greater accrual of its variability and functions [35–37]. It has been hypothesized that over one hundred CD44 isoforms can be generated, although to date only 26 have been identified. The predominant form of CD44 expressed in normal tissues does not contain any spliced exons and is designated hematopoetic or standard CD44 (CD44H or CD44s resp.). It can undergo post-translational modifications and has a molecular weight of 80–100 kDa. 

CD44 can interact with various cell surface and extracellular ligands but its principal ligand is HA [38, 39]. It is noteworthy that binding of HA to CD44 is not constitutive but is activation dependent [40]. In this respect, quiescent leukocytes express inactive forms of CD44 that do not bind to HA and must be activated before it can interact with HA. Recognition of HA by CD44 is dependent on the degree of post-translational modifications, its phosphorylation status, sulfation pattern and ability to form multivalent aggregates on the cell surface [41–44]. Binding of HA to CD44 is a relatively weak interaction in comparison to other cell receptor-ligand interactions such as those that involve integrins or cadherins, but in some instances weak interactions are an advantage particularly when leukocytes require to be in close proximity in order to exchange chemical signals prior to their activation and maturation [45, 46]. The interaction of CD44 with HA has been shown to enhance various cellular functions such as cell proliferation and migration, and activation of PKC, PI3K and MAPK-signaling pathways which have all been shown to induce inflammatory processes in autoimmune diseases including lupus nephritis [47, 48].

CD44 plays an important role in many physiological and pathological processes that include cell-cell and cell-matrix interactions, cell migration, lymphocyte activation and extravasation, and presentation of growth factors, cytokines and chemokines to their cognate receptors. Increased synthesis of CD44 and/or generation of new isoforms is often associated with pathological conditions and CD44 expression can be altered by pro-inflammatory cytokines and chemokines such as TNF-*α*, IL-1*β*, IL-8 and RANTES in both lymphoid and non-lymphoid cells. There is increasing evidence to suggest that CD44 plays a pivotal role in autoimmune diseases and its expression is increased in synovial cells in patients with rheumatoid arthritis, which correlates with synovial inflammation [49]. The administration of antibodies against CD44 can significantly reduce inflammatory processes in murine models of collagen- or proteoglycan-induced arthritis and experimental autoimmune encephalomyelitis [50–52]. CD44-HA interactions in normal murine B cells have been shown to induce cell activation, proliferation and differentiation [53]. Readers are referred to reviews by Taylor and Gallo [17] and Jiang et al. [11], which discuss the role of CD44 and HA as immune regulators during pathological disorders.

## 3. Hyaluronan and CD44 in the Pathogenesis of SLE

Alterations in the distribution pattern of HA and CD44 have been shown to play an important role in the development of SLE. Elevated serum HA levels have been observed in patients and mice with active lupus nephritis, and murine anti-dsDNA antibodies have been shown to cross-react with HA [12, 54–56]. In the next section, we will discuss the contributing role of HA and CD44 in SLE with particular emphasis of their roles in the progression of lupus nephritis.

### 3.1. HA, CD44, and Immune Cells

**Figure 2:**
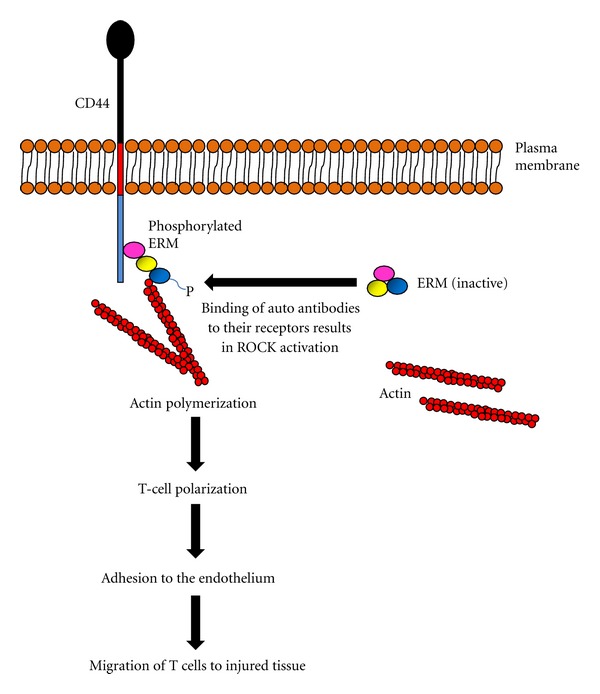
Schematic diagram showing the effect of ERM activation on T-cell function in SLE patients. Autoantibodies such as anti-CD3/T-cell receptor (TCR) antibodies bind to CD3/TCR complex in circulating T cells and induce ROCK activation, which in turn mediates ERM phosphorylation. Once activated, ERM directly interacts with CD44 and F actin resulting in their colocalization at the polar caps of T cells, leading to actin polymerization, T-cell polarization, adhesion to the endothelium and subsequent chemotactic migration to sites of injury in the kidney.

An important step in the initiation and propagation of lupus nephritis is the recruitment of immune cells, namely T cells, B cells, macrophages, and dendritic cells, to sites of injury including the kidney [57–62]. Polyclonal B-cell activation precedes the development of clinical nephritis [63], thereby highlighting the crucial role of leukocytes in the pathogenesis of disease. The mechanism of local immune regulation and leukocyte-mediated kidney injury is not well delineated and is a topic of much interest. HA can induce chemokine and cytokine secretion in both lymphoid and nonlymphoid cells and therefore assumes an important role in the activation, recruitment, and retention of lymphocytes at sites of injury [26, 64–66]. We have demonstrated that in patients with active lupus nephritis subpopulations of glomerular lymphocytic infiltrates possess cell surface HA, a finding that is not observed in healthy individuals [12]. Although the mechanism through which HA regulates the activities of immune cells in the kidney during lupus nephritis remains to be defined, studies have shown that through its interaction with CD44, HA can induce murine B-cell activation, T cell, and macrophage effector functions and dendritic cell maturation [53, 67, 68]. Siegelman et al. demonstrated that CD44-HA interactions contributes to leukocyte rolling [69], a process that is essential for their extravasation to sites of injury. These researchers further observed that a subpopulation of circulating peripheral blood T cells strongly expressed CD44-dependent adhesion in SLE patients and their existence correlated with disease activity [70]. T cells that possess increased expression of CD44 have an enhanced capacity to infiltrate the kidney and induce inflammation [71], and this is dependent on the colocalization of CD44 with F-actin and phosphorylated ezrin, radixin, and moesin (ERM) at their polar caps, resulting in their polarization and conversion from freely circulating lymphocytes to those that can adhere to the endothelium and migrate into injured tissues, a process mediated through Rho-associated, coiled coil containing protein kinase (ROCK) activation (Figure 2) [71]. Genetic deletion of CD44 or inhibition of CD44 expression using a peptide based on the CDR1 sequence of a human anti-DNA antibody inhibited lymphoproliferation in lupus-prone mice and non-autoimmune mice immunized with a monoclonal anti-DNA antibody, respectively [72, 73], thereby highlighting the importance of CD44 in the pathogenesis of SLE. Crispín et al. [74] demonstrated that CD44v3 and CD44v6 expression are increased on CD4^+^ and CD8^+^ T cells isolated from patients with SLE, which correlated with disease activity, whereas CD44v6 on T cells was associated with lupus nephritis and positivity for anti-dsDNA antibodies [74]. 

Apoptosis and the phagocytic clearance of apoptotic cells from sites of injury are tightly regulated processes that are essential for the maintenance of tissue structure and function. The recognition and removal of apoptotic bodies is mediated by macrophages. Studies have demonstrated that CD44 on the surface of macrophages plays an important role in the clearance of apoptotic bodies and this process is dependent on the prior activation of intracellular pathways such as tyrosine phosphorylation of p561ck and interaction with cytoskeletal proteins [75]. Defective clearance of apoptotic cells is a cardinal feature of SLE that results in persistent inflammation and autoimmunity, since chromatin fragments and cellular components that escape from nondigested apoptotic cells can serve as immunogens that will further exacerbate disease pathogenesis [75, 76]. Studies have demonstrated that the expression of variant CD44 isoforms is induced in activated macrophages that are present at sites of inflammation and this may alter the repertoire of CD44 ligands [77]. Furthermore, studies have demonstrated that CD44 expression is reduced on monocytes/macrophages in SLE patients, which inversely correlate with the percentage of apoptotic neutrophils [78]. Therefore, a reduction in CD44 expression together with a change in CD44 isoform on monocytes/macrophages will impair their ability to recognize and remove apoptotic cells from sites of injury. Although the mechanism through which CD44 expression is altered in SLE patients remains to be investigated, it is possible that changes in cytokine expression in the microenvironment may contribute. 

Increased expression of interferon-inducible genes is a prominent feature in SLE. Recent analysis of the interferon pathway showed an association between CD44 and SLE [79]. In a recent study, CD44 has also been linked to thrombocytopenia in SLE patients [80–82]. 

### 3.2. HA, CD44, and Resident Renal Cells

In the normal kidney, HA is found solely in the medullary and papillary interstitium of the kidney where it contributes to the mechanical stability of tubules and blood vessels, and also in the concentration of urine, whilst the expression of CD44 is restricted to passenger leukocytes and resident macrophages [83–85]. Accumulation of HA in the renal cortex is observed in patients and mice with active lupus nephritis and in autoimmune crescentic glomerulonephritis [12, 86]. *In vitro* studies have demonstrated that mesangial cells, proximal tubular epithelial cells and interstitial fibroblasts are able to synthesize HA and it is likely that these cells all contribute to the synthesis of HA in renal diseases [12, 87–91]. We and others have demonstrated that HA and CD44 expression is increased in the glomerular and tubulo-interstitial compartments of the kidneys, with predominant expression of HA and CD44 in the periglomerular area and in atrophic tubules of patients and mice with active lupus nephritis [12, 54, 85, 92, 93]. The accumulation of HA in the kidney was shown to correlate with the infiltration of lymphocytes in the tubulo-interstitium and tissue damage [92]. *In vitro* studies have shown that proinflammatory mediators involved in the pathogenesis of lupus nephritis such as TNF-*α* and IFN-*γ* can increase HA synthesis in proximal tubular epithelial cells [92], and therefore may contribute to increased synthesis of HA in lupus patients. 

We have previously demonstrated that human polyclonal anti-dsDNA antibodies can induce IL-1*β*, IL-6, and TNF-*α* in cultured human mesangial cells and proximal tubular epithelial cells [12, 94]. We further demonstrated that anti-dsDNA antibodies can induce HA synthesis in human mesangial cells and proximal tubular epithelial cells, with the production of both HMW and LMW HA, and this induction was dependent on increased synthesis of HAS II mRNA, and IL-1*β* and IL-6 secretion [12, 95]. Our observation that increased circulating HA levels in patients with lupus nephritis correlated with anti-dsDNA antibodies substantiates the likelihood that anti-dsDNA antibodies contribute to increased HA synthesis during pathogenesis of disease [12]. Considering that LMW HA possesses pro-inflammatory properties, that anti-dsDNA antibodies can induce LMW HA in resident renal cells may represent a pathogenic mechanism through which anti-dsDNA antibodies induce inflammatory processes in the kidney parenchyma during lupus nephritis. 

Exogenous LMW, but not HMW HA, has been shown to induce *de novo* synthesis of MCP-1 mRNA and protein secretion in proximal tubular epithelial cells, and this induction was dependent on the interaction of HA with CD44 [64]. Intrarenal MCP-1 expression is increased in both the glomerular and tubulo-interstitial compartments of the kidney during lupus nephritis and precedes leukocyte infiltration, proteinuria, and renal damage [96]. The importance of MCP-1 in the pathogenesis of lupus nephritis is underscored by studies by Tesch et al. [97], which demonstrated that lupus-prone mice rendered genetically deficient in MCP-1 showed less severe renal histology and proteinuria [97]. Studies have also demonstrated that exogenous LMW HA can induce ICAM-1 and VCAM-1 in murine cortical tubular epithelial cells, suggesting that HA may play a role in the adhesion of leukocytes to resident renal cells [31]. We have demonstrated that inhibition of HA synthesis in NZBWF1/J mice is associated with an improvement in clinical parameters of disease and decreased intrarenal expression of IL-6 and TNF-*α* [54].

## 4. Conclusion

Despite its simple structure, HA is a multifaceted macromolecule that, depending on its molecular weight, is involved in tissue homeostasis and pathological processes. Through its interaction with CD44, HA regulates leukocyte infiltration, secretion of inflammatory mediators, and clearance of apoptotic cells processes that dictate the severity of lupus nephritis. Although studies have demonstrated that the interaction of HA with toll-like receptors can modulate inflammatory processes in animal models of bleomycin-induced lung injury, there is currently no data on the interaction of HA and toll-like receptors in the pathogenesis of lupus nephritis. Further research into the interaction of HA with other binding proteins will provide us with a better understanding of their roles in the pathophysiology of lupus nephritis and whether targeting HA or CD44 may serve as a novel therapeutic strategy.

## Figures and Tables

**Table 1:** Functions of native and depolymerized hyaluronan.

Native hyaluronan	Hyaluronan fragments
Contributes to tissue integrity and maintenance of epithelial cell phenotype	Induces chemokine and cytokine secretion in infiltrating, renal tubular epithelial and endothelial cells
Contributes to water balance and regulation of tissue hydration	Induces phosphorylation of signaling pathways, for example, MAPK
Contributes to transportation and distribution of plasma proteins	Induces cell proliferation and migration in chondrocytes, endothelial cells and fibroblasts
Protects against tissue damage by scavenging free radicals	Activates NF*κ*B
Anti-inflammatory-can inhibit activation of inflammatory cells	Induces nitric oxide synthase
Protects against apoptosis	Suppresses cell death and apoptosis in cell culture
Anti-angiogenic	Promotes angiogenesis
Immunosuppressive-prevents ligand binding to surface receptors	Increases matrix protein synthesis, for example, collagen type I
Inhibits phagocytosis	Increases transcription of matrix metalloproteinases

Hyaluronan fragments: range from 4 to 40 saccharide units.
